# Anti-Inflammatory Effects of p-Coumaric Acid, a Natural Compound of* Oldenlandia diffusa*, on Arthritis Model Rats

**DOI:** 10.1155/2018/5198594

**Published:** 2018-02-22

**Authors:** Hao Zhu, Qing-hua Liang, Xin-gui Xiong, Yang Wang, Zhi-hui Zhang, Mei-juan Sun, Xun Lu, Dan Wu

**Affiliations:** ^1^Institute of Combined Traditional Chinese and Western Medicine, The First Affiliated Hospital of Soochow University, Suzhou, Jiangsu 215006, China; ^2^Institute of Combined Traditional Chinese and Western Medicine, Xiangya Hospital, Central South University, Changsha, Hunan 410008, China; ^3^Institute of Combined Traditional Chinese and Western Medicine, Suzhou Municipal Hospital, Soochow, Jiangsu 215008, China; ^4^Institute of Combined Traditional Chinese and Western Medicine, The 4th Hospital of Changsha, Changsha, Hunan 410008, China

## Abstract

**Objectives:**

In China,* Oldenlandia diffusa* (OD) is a natural herb that is widely used and has been proven to be effective in the treatment of rheumatoid arthritis (RA). This study aimed to preliminarily reveal the mechanism by which OD exerts its beneficial effect.

**Methods:**

Ultra-performance liquid chromatography photodiode array was applied to identify the absorbable compounds in the plasma of collagen-induced arthritis (CIA) model rats. After 2 weeks, an OD decoction or the identified absorbable compound was administered to CIA rats. Morphology, X-ray images of the joints, pathological images, arthritis index, and cytokine (TNF-*α* and IL-6) levels were evaluated.

**Results:**

p-Coumaric acid (p-CA) was identified as the absorbed compound in plasma. After administration of p-CA solution or the OD decoction, symptoms in the treated rats were alleviated as compared to the untreated model rats, and inflammatory cell infiltration was suppressed. The arthritis index and serum levels of TNF-*α* and IL-6 were decreased as compared to the control group.

**Conclusions:**

OD may exert its anti-inflammatory effect on RA via its active ingredient, p-CA. This information sheds light on the mechanism by which OD exerts its anti-inflammatory effort in RA and forms the basis for further development of therapeutic agents for RA.

## 1. Introduction

Rheumatoid arthritis (RA) is a heterogeneous systemic autoimmune disease that impairs the quality of life of patients [1] and presents with different symptoms [2]. Exterior changes involve joint malformation, swelling, and joint dysfunction [3, 4], while interior pathological changes mainly involve synovial hyperplasia and cartilage destruction. All of these changes are accompanied by systemic inflammatory responses [5, 6], involving many cytokines, such as TNF-*α* [7, 8] and IL-6 [9–11], which are responsible for initiating, propagating, and maintaining inflammation [12] in a complex network [13]. Recent studies have shown that TNF-*α* governs the crosstalk between fibroblast-like synoviocytes and chondrocytes [12] and might regulate the expressional changes in matrix-degrading enzymes and inflammatory mediators in fibroblast-like synoviocytes [12] and induce the inflammatory responses. TNF-*α* also governs the crosstalk between fibroblast-like synoviocytes and chondrocytes [12] and consequently induce inflammatory responses. IL-6 also plays a key role in RA. IL-6 can induce expression of vascular endothelial growth factor (VEGF) in synovial fibroblasts and other cells [14], promoting pannus formation in the synovium and causing an inflammatory response [14]. IL-6 is also implicated in damage to the articular cartilage in RA [15] and promotes further recruitment of leukocytes and inflammatory responses in the joints of RA patients [15]. Taken together, it is clear that inflammation plays an important role in the pathology of RA.

In China, traditional herbs are used as medication to treat patients with RA [16]. In traditional Chinese medicine, principles of yin-yang, the five elements, zang, and viscera guidelines are used [17]. Accordingly, prescriptions consisting of multiple herbs are used to treat RA patients, with many patients experiencing marked beneficial effects [18]. However, neither the mechanism of the complex traditional Chinese prescription, nor the active ingredients in these medications remain unknown; yet, such knowledge would be immensely valuable. Considering that one prescription is usually formulated from multiple herbs, research on such complex decoctions is complicated, and therefore it is convenient and advantageous to focus on a single herb when attempting to elucidate the mechanisms and active compounds.

Among the herbs traditionally used,* Oldenlandia diffusa* (OD) is a key component of herbal prescriptions for RA. Shan et al. [19] proved that OD has immunomodulating activity in vitro. Moreover, it was shown that OD could inhibit the production of TNF-*α*, IL-6, PGE-2, COX-2, and iNOS [20] and might exert anti-inflammatory effects via downregulation of TNF-*α* and IL-6 [20, 21]. These 2 cytokines are proinflammatory factors that could activate NF-*κ*B signaling [22, 23], thereby increasing the inflammatory response [24]. We have also previously shown that OD has an anti-inflammatory effect on collagen-induced arthritis (CIA) [25]. Considering that OD contains multiple ingredients, such as ursolic acid, oleanolic acid, kaempferol, p-CA, ferulic acid, rutin, scopolamine lactone, and caffeic acid [26], which of these ingredients are responsible for its effects is unknown. In this study, therefore, we investigated the active ingredients of OD further.

## 2. Methods

### 2.1. Ethics Statement

All protocols involving animals in this study were approved by the Ethics Committee of Central South University (Grant number 20120435), China. Our experiment was performed in accordance with the Guidance for the Humane Care and Treatment of Laboratory Animals published by the Ministry of Science and Technology of China.

### 2.2. Identification of Absorbable Compounds in OD

We used ultra-performance liquid chromatography (UPLC) to identify the compounds in OD that can be absorbed in rat plasma. We employed a chromatography column (ACQUITY UPLC C18; 2.1 × 50 mm; 1.7 *μ*m), at a column temperature of 38°C, wavelength of 180–360 nm, flow rate of 0.8 mL/min, and a sample volume of 8 *μ*l. Analysis time was 15 min. The reference standards (i.e., oleanolic acid, kaempferol, ursolic acid, p-CA, ferulic acid, scopolamine lactone, rutin, and caffeic acid) were purchased from Shanghai Yuanye Bio-Technology Co., Ltd., Shanghai, China. Acetonitrile and methanol (HPLC grade) were purchased from the Tedia Company, Inc. (Fairfield, OH, USA). The herbal drug OD was purchased from TCM Dispensary, Xiangya Hospital, Changsha, China. We ground OD into powder and mixed it with purified water in a ratio of 1 g of powder to 8 ml of water. After boiling for 30 min, we filtered the decoction, condensed the liquid in a vacuum, at 60°C, and dehydrated it into powder. Next, we dissolved the freeze-dried OD powder in purified water in a ratio of 1 g of powder to 10 ml of water, followed by centrifugation (1200 r/min, 15 min), after which we extracted and filtered (0.45 *μ*m filter) the supernatant. The yield rate was 43.1 mg (crude drug)/ml. We prepared the previous 8 reference standard compounds in solution by dissolving in methanol at concentrations of 0.167 mg/ml, 0.2 mg/ml, 0.163 mg/ml, 0.187 mg/ml, 0.16 mg/ml, 0.11 mg/ml, 0.168 mg/ml, and 0.232 mg/ml, respectively. We then mixed all these in a ratio of 1 : 1 : 1 : 1 : 1 : 1 : 1 : 1 to create a test solution. All solutions were sealed and then stored at a temperature of 4°C.

Sprague-Dawley (SD) rats weighing 150 ± 30 g were obtained from the Laboratory of Hunan Provincial People's Hospital. Rats were divided into 2 groups, that is, the OD group (*n* = 5) and the control group (*n* = 5). First, we calculated the standard human daily herbal dose and converted it to a rat daily herbal dose according to the body surface area of an individual weighing 70 kg [16]; the daily dose per rat was thus 2.7 mg/g (crude drug/weight). We administered the OD decoction orally at the calculated dose to the OD group and administered the same volume of purified water to the control group. Three days later, we decapitated all rats and extracted their plasma, mixed the plasma with acetonitrile, ethyl acetate, and acetone at a ratio of 1 : 2 : 1 : 0.6 (vol : vol : vol), and centrifuged the mixture (1200 r/min, 15 min). We extracted and filtered the supernatant, air dried it under nitrogen gas, redissolved and recentrifuged the sample as before, and stored it as a test sample.

### 2.3. Administration of OD and p-CA

Rats were randomly selected and divided into 2 groups: a control (*n* = 20) and a replication (*n* = 60) group, in which collagen-induced arthritis was generated. First, we prepared bovine collagen type II (BIIC) solutions with complete Freund's adjuvant and incomplete Freund's adjuvant, respectively. These 2 different solutions were subcutaneously injected into the tail, back, and soles of each rat on the 1st and 7th day, respectively. After 14 days, we randomly divided the replication group into an OD group, a p-CA group, and a model group. Based on the results of the previous chromatographic analysis, we calculated the p-CA content in OD and converted this to a rat dose [16]. Based on the normal human daily herbal dose, we calculated the daily dose of p-CA for a rat, which was 13.8 *μ*g/g. Then, we used oral gavage to administer the OD decoction (1.35 mg/g twice a day) to the OD group and p-CA solution (6.91 *μ*g/g, twice a day) for the p-CA group. The model group was given purified water (the same volume as in the other groups; twice a day). Rats in the normal group received no intervention.

### 2.4. 2.4. Morphological Analysis

On the 1st, 14th, 21st, 28th, 35th, and 42nd day, we observed and measured the arthritis index (AI) [27, 28] of each rat's posterior limb. According to the degree of joint redness and swelling, as well as joint enlargement and deformity, we scored every symptom on a scale of 0 to 4 points, with 0 meaning no arthritis, 1 meaning mild swelling plus appearance of red spots, 2 meaning moderate swelling of joints, 3 meaning severe swelling of joints, and 4 meaning severe swelling of joints and inability to bear weight.

### 2.5. Radiographic Changes in Rats' Joints

On days 0, 14, 28, and 42, X-ray images of the right rear foot of each rat were obtained to observe joint changes. We used a diagnostic X-ray machine (PHILIPS, Inc., Andover, MA, USA) to take the radiographs.

### 2.6. Pathological Changes in Rats' Joints

After injection of Freund's adjuvant with BIIC, the posterior limbs of the rats were amputated on days 14, 28, and 42. The p-CA group and OD group were divided after the 28th day, and thus the joints of these rats were amputated on days 28 and 42. All these limbs were stored in formalin solution, at 4°. Finally, we removed the synovium from each joint; all of the synovia was stored and sealed in formalin. After preparation, these samples were embedded in dehydrated paraffin and sliced into sections in 4 *μ*m and these were stained with hematoxylin-eosin (HE) as previously described [25]. Pathological changes were observed by microscope (magnified 10x; Leica DFC425C; Leica, Wetzlar, Germany).

### 2.7. TNF-*α* and IL-6 Levels

On the 28th and 42nd day, rats were decapitated and their serum was obtained. TNF-*α* and IL-6 levels were tested and compared using enzyme-linked immunosorbent assays (ELISA Cusabio Biotech Co., Ltd., Wuhan, China), as previously described [25].

### 2.8. Statistical Analyses

The data of the arthritis index and TNF-*α* and IL-6 levels were statistically analyzed using SPSS 15.0 and compared among groups.

## 3. Results

### 3.1. Identification of the Active Compounds in OD

**Figure 1:**
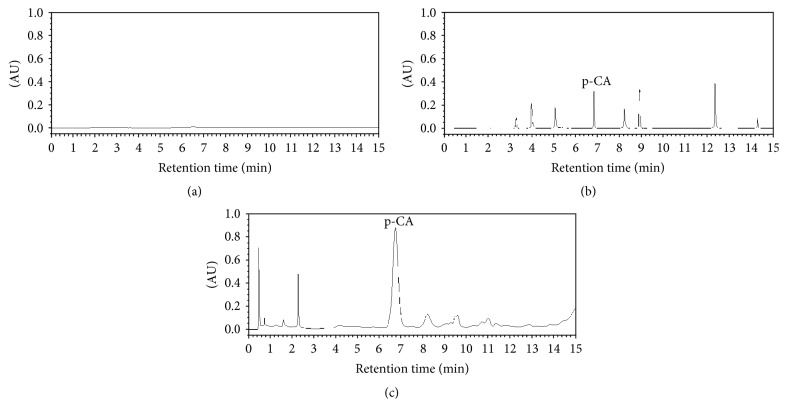
Chromatogram of each group (308 nm): (a) blank methanol; (b) 8 reference compounds; (c) test solution of OD. p-CA refers to p-coumaric acid.

In the preliminary experiment, we detected each reference separately, to find the chromatographic wavelength and showtime of each reference standard. Compared with the wavelength and showtime of each reference, as shown in Figure 1, the 8 reference standards were well separated and identified as oleanolic acid (224 nm, 3.29 min), kaempferol (366 nm, 4.02 min), ursolic acid (223 nm, 5.09 min), p-CA (308 nm, 6.81 min), ferulic acid (321 nm, 8.21 min), scopolamine lactone (228 nm, 8.92 min), rutin (255 nm, 12.36 min), and caffeic acid (325 nm, 14.23 min). When analyzing the OD solution, we observed a large peak at a wavelength of 308 nm at 6.79 min (Figure 1(c)) in the OD solution. In the blank methanol (Figure 1(a)), there was no peak around 6.79 min. We speculate that the large peak in OD group's chromatogram might be p-CA (Figure 1(c)).

### 3.2. Identification of Absorbable Compounds in OD

**Figure 2:**
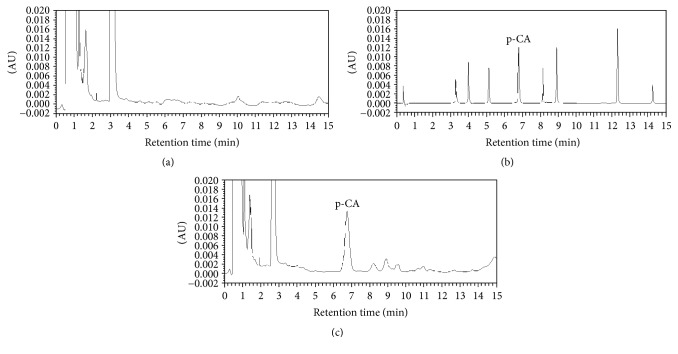
Chromatogram of each group (308 nm): (a) plasma without added compounds; (b) 8 reference compounds; (c) the plasma of rats that had been given OD decoction by oral gavage. p-CA refers to p-coumaric acid.

Compared with the wavelength and showtime of each reference, as showed in Figure 2(b), the 8 reference standards were well separated and identified as oleanolic acid (224 nm, 3.31 min), kaempferol (366 nm, 3.98 min), ursolic acid (223 nm, 5.13 min), p-CA (308 nm, 6.81 min), ferulic acid (321 nm, 8.18 min), scopolamine lactone (228 nm, 8.90 min), rutin (255 nm, 12.34 min), and caffeic acid (325 nm, 14.21 min). After orally administering the OD decoction to rats, the plasma was tested to identify which compounds were absorbed by the rats. As shown in Figure 2(c), a large peak was well separated at a wavelength of 308 nm at 6.80 min from the OD rat plasma, while blank plasma (Figure 2(a)) showed no such peak around 6.81 min. We speculate that the large peak in the plasma's chromatogram might be p-CA (Figure 2(c)).

### 3.3. Morphological Analysis

**Figure 3:**
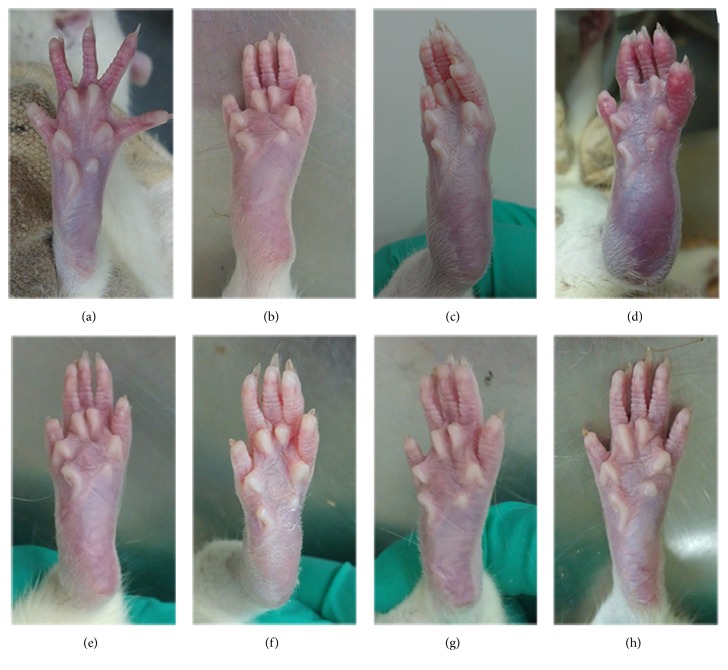
Morphological changes of joints in each group, from the 1st day to the 42nd day. (a) Posterior limb of a rat in the normal group. (b) Posterior limb of a rat in the model group, on the 14th day. (c) Posterior limb of a rat in the model group, on the 28th day. (d) Posterior limb of a rat in the model group, on the 42nd day. (e) Posterior limb of a rat treated with p-CA, on the 28th day. (f) Posterior limb of a rat treated with p-CA, on the 42nd day. (g) Posterior limb of a rat treated with OD decoction, on the 28th day. (h) Posterior limb of a rat treated with OD decoction, on the 42nd day.

Before BIIC injection, as shown in Figure 3(a), in the control group, the posterior limb remained slim and flexible. After the injection, morphological changes occurred within 4–6 days. In the model group, from the 14th day to the 42nd day (Figures 3(b)–3(d)), the ankle and toe joints of the rats gradually showed more swelling, redness, ankylosis, and hyperemia. This was particularly notable by the 42nd day (Figure 3(d)). Simultaneously, rats in the model group became inactive and drowsy and ate and drank less. After treatment with p-CA (Figures 3(e) and 3(f)), at the 28th day in the p-CA group (Figure 3(e)), there were minor changes in terms of skin shrinking and alleviation of swelling, as compared to the model group (Figure 3(c)). By the 42nd day (Figure 3(f)), ankle swelling and skin redness were notably alleviated as compared to the model group (Figure 3(d)). Similarly, after administration of the OD decoction (Figures 3(g) and 3(h)), as shown in the OD group by the 28th day (Figure 3(g)), the ankle showed a mild change in terms of skin shrinking and alleviation of swelling, as compared to the model group. By the 42nd day (Figure 3(h)), ankle swelling and skin redness were markedly alleviated as compared to the model group.

### 3.4. Radiographic Analysis of Rats' Joints

**Figure 4:**
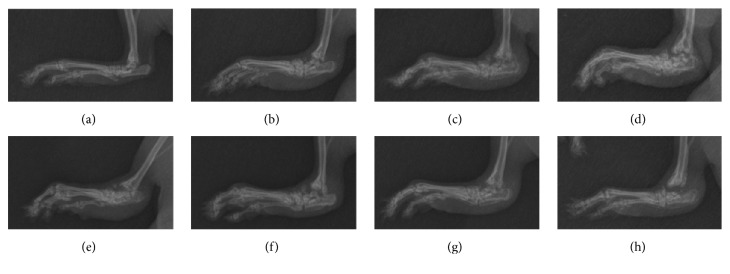
X-ray film of the joints in each group, from the 1st day to the 42nd day. (a) Posterior limb of a rat in the normal group. (b) Posterior limb of a rat in the model group, on the 14th day. (c) Posterior limb of a rat in the model group, on the 28th day. (d) Posterior limb of a rat in the model group, on the 42nd day. (e) Posterior limb of a rat treated with p-CA, on the 28th day. (f) Posterior limb of a rat treated with p-CA, on the 42nd day. (g) Posterior limb of a rat treated with OD decoction, on the 28th day. (h) Posterior limb of a rat treated with OD decoction, on the 42nd day.

X-ray images showed pathological changes inside the joints. As shown in Figure 4(a), the control group showed no swelling; both toe joints and ankle joints showed clear space. After BIIC injection, radiographic changes occurred within 4–6 days. In the model group, from the 14th day to the 42nd day (Figures 4(b)–4(d)), the toe joint space and ankle joint space gradually disappeared or became indistinct, and swelling of the surrounding tissue gradually worsened. By the 42nd day (Figure 4(d)), some toe joints even showed joint fusion and loss of function. After administration of p-CA, from the 28th day to the 42nd day (Figures 4(e) and 4(f)), the space in the ankle joint of the p-CA group gradually became clear as compared to the model group (Figures 4(c) and 4(d)). After treatment with OD decoction, from the 28th day to the 42nd day (Figures 4(g) and 4(h)), the same radiographic changes as seen in the p-CA group were even more obvious in the OD group, as compared to the model group (Figures 4(c) and 4(d)). The tissue swelling in the p-CA and OD group by the 42nd day (Figures 4(f) and 4(h)) showed marked alleviation, as compared with the model group (Figure 4(d)). All posterior limbs were functional by the 42nd day after administration of p-CA or OD decoction (Figures 4(f) and 4(h)). The p-CA and OD groups thus showed a considerable amelioration of arthritic ankle joints.

### 3.5. Pathologic Changes in Rats' Joints

**Figure 5:**
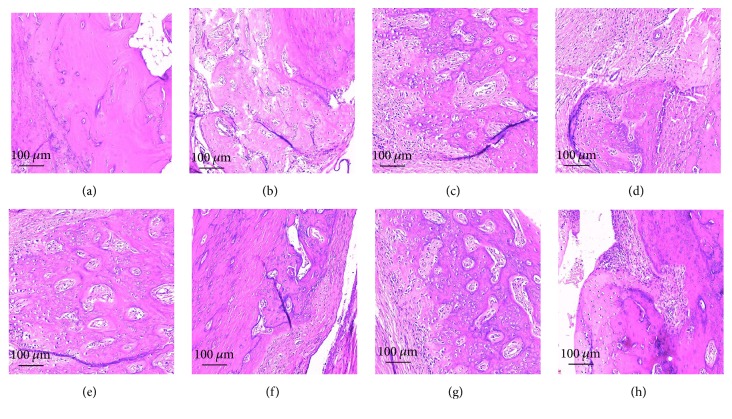
Synovium pathomorphology as seen by hematoxylin-eosin-staining in each group, from the 1st day to the 42nd day. (a) Normal group. (b) Model group, 14th day. (c) Model group, 28th day. (d) Model group, 42nd day. (e) p-CA group, 28th day. (f) p-CA, 42nd day. (g) OD group, 28th day. (h) OD group, 42nd day.

As shown in Figure 5(a), mild inflammatory cell infiltration was observed in the control group. As compared with the control group, the inflammatory cell infiltration deteriorated from the 14th day (Figure 5(b)) to the 42nd day (Figure 5(d)) in the model group. The infiltration became more concentrated during the course in the model group and was particularly notable on the 28th day (Figure 5(c)) and 42nd day (Figure 5(d)). We then treated the p-CA group on the 14th day; 2 weeks later, that is, on the 28th day, as shown in Figure 5(e), there was no major change in inflammatory cell infiltration, as compared with the model group at the time point (Figure 5(d)). After another 2 weeks, that is, at the 42nd day, in the p-CA group (Figure 5(f)), inflammatory cell infiltration was alleviated as compared with the model group (Figure 5(d)), as well as with the inflammatory cell infiltration 2 weeks earlier (Figure 5(e)). Two weeks after treatment of the OD group on the 14th day, that is, on the 28th day, as shown in Figure 5(g), there was no major change in inflammatory cell infiltration as compared with the model group at the time (Figure 5(c)). After another 2 weeks, that is, on the 42nd day, the inflammatory cell infiltration in the OD group (Figure 5(h)) was alleviated as compared with the model group (Figure 5(d)), as well as with the infiltration 2 weeks earlier (Figure 5(g)). 

### 3.6. Analysis of Arthritis Index

Before intervention, as shown in Figure 5, each group was scored as 0 on the AI. The control group had a score of 0 throughout the experiment. After injection, from the 14th day to the 21st day, the index of the other 3 groups started to increase and was not significantly different among the model, p-CA, and OD groups. From the 28th day to the 42nd day, the index of the model group stabilized at a high value, whereas both the p-CA group and the OD group had lower index values than the model group (*p* < 0.05), which continuously decreased from the 21st day to the 42nd day. On the 28th day, the index of the OD group appeared to be lower than that of the p-CA group, but without statistical significance. On the 35th and particularly on the 42nd day, the OD group had a lower AI than the p-CA group (*p* < 0.05).

### 3.7. Analysis of TNF-*α* and IL-6 Levels

**Figure 6:**
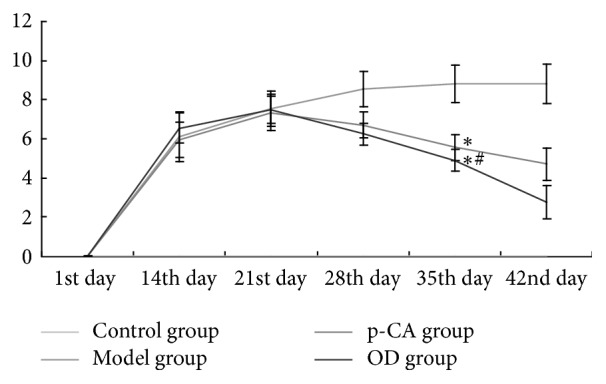
Arthritis index (AI) in each group (means ± SD), from the 1st day to the 42nd day. From the 28th day to the 42nd day, the p-CA group and OD group showed statistically significant differences (^*∗*^*p* < 0.05) as compared to the model group. On the 42nd day, the OD group showed a significant difference (^#^*p* < 0.05) as compared with the p-CA group.

According to the ELISA results shown in Figure 6, TNF-*α* levels in the model group rose from the 28th day to the 42nd day and were markedly higher than those in the control group. After intervention, on both the 28th day and the 42nd day, the p-CA group had lower levels than the model group (*p* < 0.05), although these values were still higher than those in the control group. The level of TNF-*α* in the OD group decreased from the 28th day to the 42nd day and was lower than those in the model group (*p* < 0.05). Additionally, as shown in Figure 6, the IL-6 levels in the model group increased from the 28th day to the 42nd day and were markedly higher than those in the control group. After intervention, on both the 28th day and the 42nd day, the levels in the p-CA group were lower than those in the model group (*p* < 0.05) but remained higher than those in the control group. The level of IL-6 in the OD group decreased from the 28th day to the 42nd day and was lower than those in the model group (*p* < 0.05).

## 4. Discussion

In China, OD has been widely used for treating RA, and multiple components in OD, including p-CA, have been identified [29, 30]. p-CA is one of the active ingredients in OD and could prevent cell-mediated immune responses in rats [31] and decrease the expression of the inflammatory mediators TNF-*α* and IL-6 [31] and circulating immune complexes in adjuvant-induced arthritic rats [31]. Using UPLC, we verified that p-CA is one of the ingredients in OD (Figure 1) and that it could be absorbed into rat's blood after intragastric administration of OD decoction (Figure 2). Based on these findings, we administered p-CA and OD, separately, to CIA model rats. As shown in Figures 3 and 4, CIA led to a range of inflammatory features, including joint swelling, hyperemia, joint fusion, and loss of function. After treatment with p-CA or OD, these symptoms were alleviated, including skin shrinking, alleviation of swelling, clearing of the joint space, and improvement of the range of motion. Thus, p-CA and OD could alleviate CIA symptoms. The change in AI (Figure 6) also demonstrated that both p-CA and OD could downregulate CIA symptoms. These findings strongly indicate that OD might alleviate CIA symptoms via its ingredient, p-CA.

**Figure 7:**
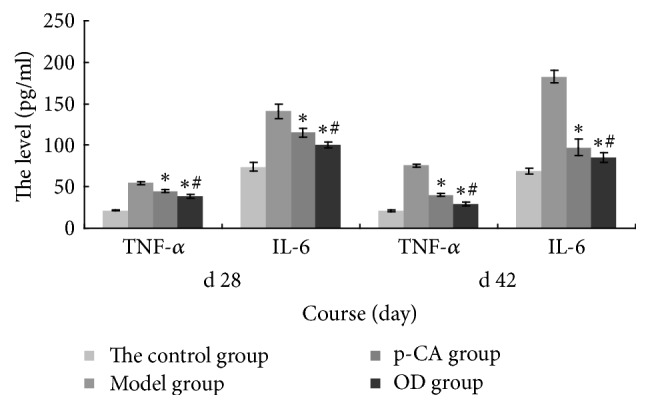
Serum levels of TNF-*α* and IL-6 in each group (means ± SD) on the 28th day and 42nd day. The p-CA group and OD group showed statistically significant differences (^*∗*^*p* < 0.05) as compared with the model group. The OD group also showed a significant difference (^#^*p* < 0.05) as compared with the p-CA group.

Subsequent experiments shed light on how p-CA or OD exerted these effects. TNF-*α* and IL-6 are well-known cytokines that play important roles in the pathogenesis of RA [32]. TNF-*α* is released from macrophagocytes in the synovium and pannus and may have potent antioxidant and anti-inflammatory effects [33]. IL-6 can trigger the immune system by activating B-cells [34], releasing immunoglobulins, and increasing the production of rheumatoid factor [35, 36]. It could also enhance the production of TNF-*α* and IL-1*β* [15], thereby aggravating cartilage destruction [15, 37]. Recent studies have shown that TNF-*α* and IL-6 could induce oxidative stress by initiating the NF-*κ*B activation cascade [38], causing erosion of the articular cartilage as well as bone destruction [39, 40]. In this study, we showed that the serum levels of TNF-*α* and IL-6 increased markedly in the CIA rat model of RA, and after treatment with p-CA or OD these cytokine levels decreased (Figure 7). This may indicate that both p-CA and OD could suppress TNF-*α* and IL-6 and thereby could counter the inflammatory response, such as inflammatory cell infiltration [41]. As shown in Figure 4, the model group had more marked inflammatory cell infiltration than the control group, which was related to the high serum levels of TNF-*α* and IL-6, and after intervention with p-CA or OD the cell infiltration in both groups was alleviated. Thus, both p-CA and OD could suppress the infiltration of inflammatory cells, supporting its anti-inflammatory effect. A previous report also indicated that p-CA could have a moderate inhibitory effect on the activation of NF-*κ*B [42], which could accelerate vascular proliferation, synovial hyperplasia [43], and cytokine proliferation [44, 45]. Pragasam et al. [31] also showed that p-CA may inhibit the expression of the NF-*κ*B gene, resulting in reduced TNF-*α* expression. These results are concordant with our own.

## 5. Conclusion

Our study thus indicated that both p-CA and OD could alleviate the symptoms of arthritis in rats. Considering that p-CA is one of the active ingredients in OD, we speculated that OD may exert its anti-inflammatory effects via p-CA, in a mechanism that included suppression of inflammatory cell infiltration, as well as the levels of TNF-*α* and IL-6. Given that OD contains multiple ingredients, we speculate that there are additional effective ingredients that play a role in the therapeutic effect of OD, which may have additional targets. Elucidating the mechanisms underlying the effects of OD in RA will therefore require further study. This study has shed light on the mechanism by which OD exerts its anti-inflammatory effort in RA and forms the basis for further development of therapeutic agents for RA.

## Abbreviations

AI: Arthritis index
BIIC: Bovine collagen type II
CIA: Collagen-induced arthritis
OD: * Oldenlandia diffusa*
p-CA: p-Coumaric acid
RA: Rheumatoid arthritis
UPLC-PDA: Ultra-performance liquid chromatography photodiode array.
